# Association between Gastric Cancer and Osteoporosis: A Longitudinal Follow-Up Study Using a National Health Sample Cohort

**DOI:** 10.3390/cancers16132291

**Published:** 2024-06-21

**Authors:** Kyeong Min Han, Mi Jung Kwon, Joo-Hee Kim, Ji Hee Kim, Woo Jin Bang, Hyo Geun Choi, Dae Myoung Yoo, Na-Eun Lee, Nan Young Kim, Ho Suk Kang

**Affiliations:** 1 Hallym Data Science Laboratory, Hallym University College of Medicine, Anyang 14068, Republic of Korea; km.han@hallym.ac.kr (K.M.H.); ydm@hallym.ac.kr (D.M.Y.); d23009@hallym.ac.kr (N.-E.L.); 2 Department of Pathology, Hallym University Sacred Heart Hospital, Hallym University College of Medicine, Anyang 14068, Republic of Korea; mulank99@hallym.or.kr; 3 Division of Pulmonary, Allergy, and Critical Care Medicine, Department of Internal Medicine, Hallym University Sacred Heart Hospital, Hallym University College of Medicine, Anyang 14068, Republic of Korea; luxjhee@hallym.or.kr; 4 Department of Neurosurgery, Hallym University Sacred Heart Hospital, Hallym University College of Medicine, Anyang 14068, Republic of Korea; kimjihee@hallym.or.kr; 5 Department of Urology, Hallym University Sacred Heart Hospital, Hallym University College of Medicine, Anyang 14068, Republic of Korea; yybbang@hallym.or.kr; 6 Suseo Seoul E.N.T. Clinic, 10, Bamgogae-ro 1-gil, Gangnam-gu, Seoul 06349, Republic of Korea; mdanalytics@naver.com; 7 Hallym Institute of Translational Genomics and Bioinformatics, Hallym University Medical Center, Anyang 14068, Republic of Korea; honeyny@hallym.or.kr; 8 Division of Gastroenterology, Department of Internal Medicine, Hallym University Sacred Heart Hospital, Hallym University College of Medicine, Anyang 14068, Republic of Korea

**Keywords:** gastric cancer, osteoporosis, nested case–control study, national healthcare data

## Abstract

**Simple Summary:**

Using data from the Korean National Health Insurance Service—National Sample Cohort database, we evaluated whether patient-specific factors such as age, sex, income, residence, and the presence of comorbidities influence the relationship between gastric cancer (GC) and the likelihood of developing osteoporosis. Our study, with 1:4 propensity score matching and a stratified Cox proportional hazards model, showed that the hazard ratio of osteoporosis for the GC group was 1.13 (95%CI, 1.03–1.24) compared to that for the control group. In addition, subgroup analysis showed that age < 65 years, male sex, and a Charlson Comorbidity Index of 0 were risk factors for osteoporosis.

**Abstract:**

Gastric cancer (GC) survivors may be more likely to develop osteoporosis. However, few studies on the relationship between GC and osteoporosis have been conducted on large patient populations. We aimed to determine the incidence of osteoporosis and identify related factors by comparing patients with GC and matched controls using the Korean National Health Insurance Service—National Sample Cohort (KNHIS-NSC). This study included 9078 patients with GC and 36,312 controls (1:4 propensity score-matched for sex, age, residence, and income). The hazard ratio (HR) for osteoporosis was significantly greater for GC patients than for controls according to Charlson Comorbidity Index (CCI) score-adjusted models (adjusted HR = 1.13). Kaplan–Meier analysis revealed that the cumulative incidence of osteoporosis during the follow-up period commencing from the index date was significantly greater in GC patients than in the controls (*p* = 0.0087). A positive correlation of osteoporosis with GC was detected for those aged < 65 years, males, and those with CCI scores = 0. In conclusion, the study findings suggest that men with GC aged < 65 years may be at an increased risk for osteoporosis. Research into additional risk factors and the optimal timing of interventions are needed to prevent fractures and minimize bone loss in GC survivors.

## 1. Introduction

According to Global Cancer Statistics 2020, with more than 1 million estimated new cases worldwide, gastric cancer (GC) is the fifth most common malignancy [1]. Recently, there has been an increase in the GC incidence rate among people under 50 years of age in both low- and high-incidence countries, especially among men [2]. In Korea, with the universalization of gastroduodenoscopy through the GC screening program, more than half of GC cases are detected at an early stage [3]; the overall survival rate exceeds 67% for GC patients and is 92% for early-stage GC patients [4]. This result is not much different from the results of Japan, a fellow East Asian country [5]. Therefore, effectively managing health issues following treatment for GC in patients who are diagnosed early and fully cured is also a critical concern.

Osteoporosis is a progressive skeletal disease characterized by decreased bone density along with bone fragility and collateral damage to the bone microstructure, which can lead to osteoporotic fractures and decrease quality of life. Meta-analyses have shown that a lower bone mineral density (BMD) is associated with a significantly increased risk of death due to all causes [6]. After gastrectomy for GC, calcium and vitamin D absorption are reduced due to decreased gastric acid secretion and rapid passage of food through the small intestine [7]. Absorbed calcium and vitamin D are important for calcium–phosphorus homeostasis, and vitamin D deficiency can cause bone loss, osteoporosis, and fractures [8]. Additionally, postoperative weight loss, low physical activity, and malnutrition are risk factors for osteoporosis [9,10,11,12]. As a result, the incidence of osteoporosis after gastrectomy for GC is 32–42% [13], and the risk of osteoporosis and hip fractures in survivors after gastrectomy is 3.7 and 1.8 times greater than that in healthy controls, respectively [14,15].

For GC patients with general risk factors for osteoporosis, such as a low-trauma fracture history, postmenopausal females, and males over 50 years of age, the American Gastroenterological Association (AGA) recommends dual-energy X-ray absorptiometry (DXA) examination 10 years after GC surgery [13]; however, the AGA guidelines state that more studies are needed to discover other risk factors for the development of osteoporosis and conduct more efficient screening with techniques such as DXA [13]. Several studies on GC and osteoporosis or osteoporotic fractures have indicated that female sex, old age, and low body mass index are risk factors for developing osteoporosis. However, most studies were conducted at a single center, with a relatively small number of patients [12,16,17,18,19]. Some studies included only men [20] or did not use matched controls [12,16,18,21]. Therefore, few studies have systematically evaluated the risk factors for osteoporosis in a large cohort by comparing patients treated for GC and matched controls.

In this study, we aimed to determine the incidence of osteoporosis and identify related factors by comparing GC patients and healthy controls using the Korean National Health Insurance Service—National Sample Cohort (KNHIS-NSC).

## 2. Materials and Methods

### 2.1. Data Source

The Ethics Committee of Hallym University (IRB No. 2022-10-008) approved this study, and written informed consent was waived because the analysis was performed using anonymous retrospective data. All analyses in this study adhered to the guidelines and regulations of the ethics committee of Hallym University. The data utilized during this retrospective cohort study were sourced from the KNHIS-NSC while ensuring individual privacy through an identification prevention process. The KNHIS covers more than 98% of the population of Korea through an obligatory policy, and the KNHIS-NSC comprises 1,137,861 patients and 219,673,817 medical claim codes documented between January 2002 and December 2019 [22].

### 2.2. Participant Selection

We selected GC cases from the KNHIS-NSC database between 2005 and 2019 (*n* = 10,174). The controls were defined as those who were not diagnosed with GC between 2002 and 2019 (*n* = 1,127,687). Among the controls, patients who were diagnosed with GC at least once before 2002 or after 2019 were excluded (*n* = 2412).

**Figure 1:**
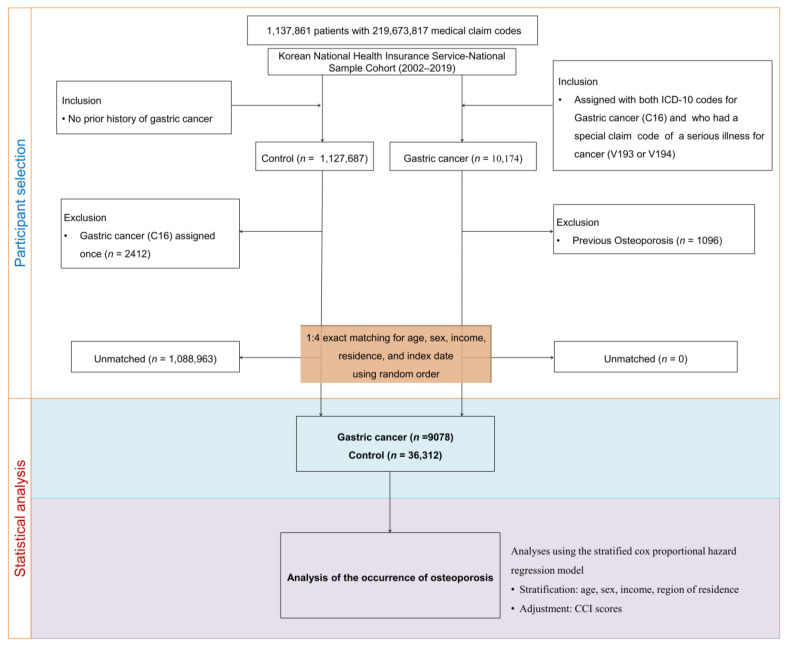
Schematic illustration of the participant selection process. Out of the initial pool of 1,127,681 patients, 10,174 gastric cancer cases were carefully matched with 36,312 controls based on factors including age, sex, income, and residence.

To adjust the balance of baseline characteristics between cases and controls, we stratified gastric cancer (GC) cases and controls into 360 layers based on age (18 age groups at 5-year intervals: 40–44 years, 45–49 years, …, 85+ years), sex (male/female), monthly income level (five income groups: class 1 [lowest income] to class 5 [highest income]), and residence area (urban: Seoul, Busan, Daegu, Incheon, Gwangju, Daejeon, and Ulsan; rural: Gyeonggi Province, Gangwon Province, North and South Chungcheong Provinces, North and South Jeolla Provinces, North and South Gyeongsang Provinces, and Jeju Province). Each layer was matched at a 1:4 ratio; during the matching process, all controls that did not completely match GC cases were excluded. We limited selection bias by arranging controls using an order indicated by randomly assigned numbers and then selecting matched participants from the top of the list to the bottom. The date on which ICD-10 code C16 was recognized in health insurance claims datasets was taken as the index date for GC participants. Both GC cases and controls had the same index date. Patients who had a history of osteoporosis before the index date were excluded; for this reason, 1096 participants were excluded from the GC group (left-truncated). Finally, 1,088,963 controls were excluded during the matching procedure, and 9078 GC cases were 1:4 matched with 36,312 controls (Figure 1).

### 2.3. Definition of Variables

#### 2.3.1. GC (Exposure)

GC cases were identified using the ICD-10 code C16. To remove false-positive GC cases, cases were further selected based on the existence of special claims codes (V193 or V194). These special codes can only be granted if a person has been certified as a cancer patient by the Korean government; these special claim codes have been settled by the government since 2005, and we selected GC patients from the KNHIS-NSC data between 2005 and 2019 (*n* = 10,174).

#### 2.3.2. Definition of Osteoporosis (Outcome)

Osteoporosis patients was defined as those assigned the ICD-10 codes M80 (osteoporosis with pathologic fracture), M81 (osteoporosis without pathologic fracture), or M82 (osteoporosis with disease classified elsewhere). Among these osteoporosis patients, those who visited the medical clinic more than twice from 2002 to 2019 and were diagnosed with osteoporosis through bone density testing using X-ray or CT (claim codes: E7001-E7004 or HC341-HC345) were selected.

#### 2.3.3. Covariates

Participants were classified into 18 age groups, 5 income groups, and 2 residential regions using the same criteria used for matching [23]. The Charlson Comorbidity Index (CCI) is a widely used index to quantify comorbid diseases burden using 17 potential comorbidities (cerebral vascular accident, acute myocardial infarction, congestive heart failure, peripheral vascular disease, pulmonary disease, connective tissue disorder, dementia, paraplegia, peptic ulcer, liver disease, severe liver disease, diabetes, diabetes complications, renal disease, cancer, metastatic cancer, and HIV) [24]. In this study, on excluding the CCI score by GC, the CCI score was scored from 0 to 29 according to the number and severity of comorbidities.

### 2.4. Statistical Analyses

**Figure 2:**
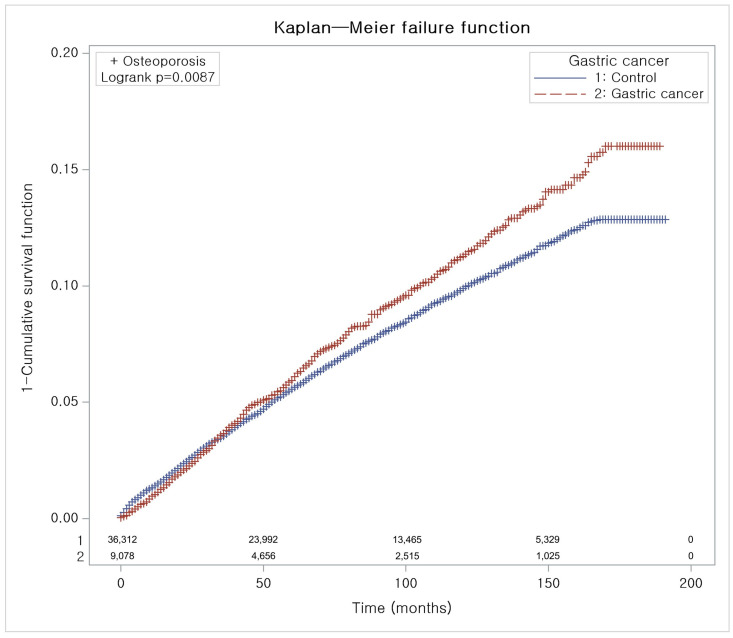
Kaplan–Meier probability of the incidence of osteoporosis in the gastric cancer cases and in controls during the 2005–2019 period from the index date.

To mitigate selection bias, 1:4 matching was performed based on age, sex, income, and residence [25]. In this process, matching according to comorbidity such as CCI score was not implemented. Next, we compared general characteristics between groups using standardized differences (Table 1); values ≤ 0.20 indicated a good balance for a particular covariate. A stratified Cox proportional hazards model was used to analyze the hazard ratios (HRs) of osteoporosis for GC, and 95% confidence intervals (95%CIs) were calculated. Crude and adjusted models were used for analysis. The crude model is a stratified Cox analysis using four variables (age, sex, income, and residence) for matched GC and control groups. The adjusted model referred to the crude model further adjusted with the CCI score. Subgroup analyses were conducted using a stratified Cox proportional hazard model for matched variables and an unstratified Cox proportional hazard model for unmatched variables (Table 2): we classified participants by age (<65 and ≥65 years old), sex (male and female), income level (highest and lowest), region of residence (urban and rural), and CCI score (0, 1, and ≥2). Osteoporosis crude incidence rates (IRs) and incidence rate differences (IRDs) were calculated by dividing the number of cases by the total number of person-years at risk and are presented per 1000 person-years. To compare the incidence of osteoporosis between groups, the Kaplan–Meier (KM) method using log-rank tests was used (Figure 2). Two-tailed *p* < 0.05 was considered statistically significant. All analyses were performed using SAS 9.4 software (SAS Institute Inc., Cary, NC, USA).

## 3. Results

### 3.1. Baseline Characteristics

The baseline characteristics of 9078 GC cases and 36,312 matched controls are shown in Table 1. For both groups, baseline characteristics were well balanced (standardized difference of 0.00) after meticulous matching in terms of age, sex, income, and residence. There was no meaningful difference (standardized difference of 0.02) in the proportions of patients with osteoporosis between the GC and control groups (6.57% and 7.17%, respectively). However, the CCI scores were imbalanced between the GC cases and controls, resulting in a standardized difference of 0.67.

### 3.2. Osteoporosis HRs in Relation to GC

As shown in Table 2, the HRs for osteoporosis were significantly greater in the GC cases than those in the controls according to both the crude and CCI score-adjusted models (crude HR = 1.16; 95%CI = 1.06–1.27; *p* = 0.001; adjusted HR = 1.13; 95%CI = 1.03–1.24; *p* = 0.01). KM analysis using the log-rank test revealed that the cumulative incidence of osteoporosis during the follow-up period commencing from the index date was significantly greater in GC cases than in the controls (*p* = 0.0087, Figure 2).

### 3.3. Subgroup Analysis

We further conducted subgroup analyses stratified by multiple covariates. A positive correlation of osteoporosis with GC was detected for individuals aged < 65 years (adjusted HR = 1.28; 95%CI = 1.11–1.48; *p* < 0.001), males (adjusted HR = 1.24; 95%CI = 1.07–1.45; *p* < 0.005), high-income individuals (adjusted HR = 1.14; 95%CI = 1.01–1.30; *p* = 0.031), and rural residents (adjusted HR = 1.17; 95%CI = 1.04–1.31; *p* = 0.01). According to the CCI scores, a CCI of 0 was positively correlated with osteoporosis and GC (adjusted HR = 1.34; 95%CI = 1.16–1.55; *p* < 0.001); however, CCIs of 1 (adjusted HR = 0.94; 95%CI = 0.76–1.16; *p* = 0.553) and ≥2 (adjusted HR = 1.08; 95%CI = 0.94–1.25; *p* = 0.283) were not significantly correlated with osteoporosis and GC.

## 4. Discussion

This is a rare study that compared the incidence of osteoporosis in GC patients who underwent gastrectomy with that in a well-matched general population. As a result of this study, HRs for osteoporosis were significantly greater in GC cases than in controls (adjusted HR = 1.13; 95%CI = 1.03–1.24; *p* = 0.01). The risk of developing osteoporosis in GC cases was more pronounced in the following groups: males, those under 65 years of age, urban residents, high income, and low CCI.

Studies conducted on large patient populations on the relationship between GC and osteoporosis are scarce. In a study by Shin et al., 133,179 GC survivors were matched 1:1 with noncancer controls for age, sex, residence, income, and disability. Compared with the matched controls, GC patients had a greater risk of osteoporotic fracture (HR = 1.61; 95%CI = 1.53–1.70), and total gastrectomy, adjuvant chemotherapy, and anemia were identified as risk factors for osteoporotic fracture [26]. However, they identified risk factors among GC patients, excluding those in the control group. Yun et al. examined osteoporotic fractures in 7082 GC surgery survivors and used a control group of 21,246 people using 1:3 propensity score matching for sex, age, and the presence of medical insurance [27]. They analyzed men and women separately and found that both men (HR = 1.13; 95%CI = 1.00–1.27) and women (HR1.18; 95%CI = 1.06–1.30) among GC surgery survivors had an increased risk of fracture compared to that in the general population. For men, low income (HR = 1.19; 95%CI = 1.04–1.36), a medically vulnerable area (HR = 1.24; 95%CI = 1.08–1.41), and a CCI score ≥ 3 (HR = 1.33; 95%CI = 1.15–1.54) were risk factors for osteoporotic fracture. For women, a CCI score ≥ 3 (HR = 1.16; 95%CI = 1.03–1.32) was a risk factor. However, in Yun’s study, there were insufficient data on whether the GC survivor group and control group were well balanced according to sex, age, medical insurance, residence, and income. However, risk factors were identified among all included patients regardless of whether they had GC. Similar to the two studies above, our study used large-scale population-based data to create a balanced comparison group without GC broadly matched on a variety of sociodemographic variables. However, unlike the above studies, when conducting subgroup analyses, we looked at the differences in age, sex, residence, income, and CCI score between the GC group and the control group. In the GC group, age < 65 years (adjusted HR = 1.28; 95%CI = 1.11–1.48; *p* < 0.001), male sex (adjusted HR = 1.24; 95%CI = 1.07–1.45; *p* < 0.005), and CCI score = 0 (adjusted HR = 1.34; 95%CI = 1.16–1.55; *p* < 0.001) were confirmed to be risk factors for osteoporosis.

In general, the prevalence of osteoporosis in women is known to be greater than that in men, and a survey of Koreans over the age of 50 years revealed that the prevalence was more than five times greater in women than in men [28]. According to a meta-analysis of GC patients after surgery, the incidence of osteoporosis was significantly greater (odds ratio = 1.90) in women than in men [29]. However, this meta-analysis was not a comparison of the occurrence of osteoporosis in GC patients and a healthy control group but rather a comparison of men and women after GC surgery. According to a prospective study targeting only men, the BMD and T score after gastrectomy for GC decreased significantly as early as 1 year after gastrectomy [12]. Another study revealed that the incidence of osteoporotic fractures after gastrectomy was 48.7 and 30.1 per 1000 person-years in men and women, respectively, with a significantly greater incidence in men [18]. In our study of all age groups, the incidence of osteoporosis in GC patients was 3.30% among men and 17.12% among women. The prevalence among all age groups was lower than the previously known prevalence rate, but the prevalence ratio between men and women was similar to that in previous studies. Due to differences in endocrinological factors, physical activity, diet, and smoking status [18], men’s BMD peaks in their 30s and then gradually decreases, while women’s BMD is maintained until their 40s and then rapidly decreases in their 50s [30]. In general, the osteoporosis screening rate in the general population is low, at 56% for women and 38% for men [31], and 88.4% of GC survivors are not aware of their bone health status [32]. In addition, compared to healthy controls, GC patients under 65 years of age or with a CCI score of 0 had a significantly greater risk of developing osteoporosis in our study. Currently, there are very few clinical guidelines for osteoporosis prevention in postgastrectomy patients. The AGA guidelines, published in 2003, state that risk factors for osteoporosis are postmenopausal females, males aged > 50 years, and patients with low-trauma fractures. Therefore, based on recent studies [12,18] and our current study, risk factors such as age < 65 years, male sex, and CCI score should be validated through additional research, and these individual risk factors should be reflected in the guidelines. A meta-analysis of 19 cohort studies showed that osteoporosis and fractures occur relatively early (at least 5 years) after gastrectomy [29]. In a study of 39 men, it was confirmed that BMD significantly decreased 1 year after gastrectomy [12], and in another study, the fracture rates were 9.1%, 19.7%, and 37.3% at 2, 4, and 5 years after surgery, respectively [18]. As shown in Figure 2 of this study, as a result of 10 years of follow-up, it was confirmed that the osteoporosis diagnosis rate among GC patients is significantly different from that among the controls, and this difference begins to appear from the third to fourth years. The AGA guidelines recommend measuring bone mineral density in gastrectomy patients after 10 years [13]. However, considering the importance of maintaining bone density after adulthood, research is needed on the effectiveness of bone density testing within 10 years after GC treatment. Additionally, prospective studies should be conducted on the usefulness of pre-emptive treatment such as vitamin D and calcium supplementation to prevent osteoporosis in GC patients.

Our study has several limitations. First, in our study, due to a lack of data, we did not classify the risk of osteoporosis in GC survivors according to the treatment modality (gastrectomy or endoscopic treatment). According to a study of 85,124 GC survivors using KNHIS and Korea Central Cancer Registry data, the ratio of surgery to endoscopy was approximately 2:1 in Republic of Korea [33], and the risk of osteoporosis in the gastrectomy group was found to be 1.23 times greater than that in the endoscopic treatment group [33]. However, osteoclastogenesis and osteoclast activity can be influenced by proinflammatory cytokines, such as tumor necrosis factor-α, which play important roles in inflammatory, infectious, and tumor processes [34,35]. Vitamin D can play a role in inhibiting the viability, proliferation, and metastasis of GC cells and inhibiting *Helicobacter pylori* infection, so vitamin D deficiency may be a risk factor for both osteoporosis and GC [36,37]. Additionally, proton pump inhibitors, which are commonly used before and after endoscopic treatment for GC, are risk factors for osteoporosis [38]. Therefore, it is possible that the increased severity of osteoporosis in GC patients is caused not only by surgical treatment but also as a byproduct of GC itself and the treatment process, and the significance of our study can be viewed from this perspective. Second, despite the evaluation of adjusted hazard ratios of osteoporosis incidence in GC according to the CCI score, this study did not include sufficient GC cases for stratification and analysis according to each comorbid disease. Another drawback of our study is that we were unable to collect information on proton pump inhibitor usage, *H. pylori* infection status, body mass index, cancer stage, and smoking history, which may be risk factors for osteoporosis. Furthermore, we did not examine the potential influence of lifestyle confounding factors that are thought to be associated with bone mass growth and maintenance, such as regular physical activity or adequate nutrition including calcium, vitamin D, protein, and vegetable intake [39,40]. In particular, it is necessary to consider the effect of vitamin D and calcium supply on osteoporosis after GC gastrectomy [41]. Moreover, regarding the various risk factors identified in our study, it would be interesting for future studies to conduct genome-wide correlation studies and studies using various omics technologies individually to confirm the relationship between GC and osteoporosis [42]. However, our study has the advantage of comparing basic characteristics such as age, sex, income, residence, and underlying disease as much as possible and making balanced comparisons between the two groups.

## 5. Conclusions

Our findings suggest that age < 65 years, male sex, and CCI scores = 0 are associated with an increased risk of osteoporosis in GC patients. Further studies are needed to discover additional risk factors and determine when interventions should be implemented to prevent future fractures and minimize bone loss in GC survivors.

## Figures and Tables

**Table 1:** Baseline characteristics of the participants.

Characteristics		Total Participants		
		Gastric Cancer (n = 9078)	Control (n = 36,312)	Standardized Difference
Demographic characteristics				
Age (n, %)				0.00
	0–4	N/A	N/A	
	5–9	1 (0.01)	4 (0.01)	
	10–14	3 (0.03)	12 (0.03)	
	15–19	N/A	N/A	
	20–24	1 (0.01)	4 (0.01)	
	25–29	19 (0.21)	76 (0.21)	
	30–34	94 (1.04)	376 (1.04)	
	35–39	204 (2.25)	816 (2.25)	
	40–44	463 (5.10)	1852 (5.10)	
	45–49	700 (7.71)	2800 (7.71)	
	50–54	969 (10.67)	3876 (10.67)	
	55–59	1149 (12.66)	4596 (12.66)	
	60–64	1350 (14.87)	5400 (14.87)	
	65–69	1314 (14.47)	5256 (14.47)	
	70–74	1246 (13.73)	4984 (13.73)	
	75–79	835 (9.20)	3340 (9.20)	
	80–84	499 (5.50)	1996 (5.50)	
	85+	231 (2.54)	924 (2.54)	
Sex (n, %)				0.00
	Male	6616 (72.88)	26,464 (72.88)	
	Female	2462 (27.12)	9848 (27.12)	
Income (n, %)				0.00
	1 (lowest)	1733 (19.09)	6932 (19.09)	
	2	1138 (12.54)	4552 (12.54)	
	3	1473 (16.23)	5892 (16.23)	
	4	1946 (21.44)	7784 (21.44)	
	5 (highest)	2788 (30.71)	11,152 (30.71)	
Region of residence (n, %)				0.00
	Urban	3892 (42.87)	15,568 (42.87)	
	Rural	5186 (57.13)	20,744 (57.13)	
Medical history				
CCI score (Mean, SD)		2.42 (2.72)	0.99 (1.69)	0.64
Osteoporosis (n, %)		596 (6.57)	2602 (7.17)	0.02

Abbreviations: CCI, Charlson comorbidity index; N/A, not applicable.

**Table 2:** Crude and adjusted hazard ratios (95% confidence intervals) of osteoporosis incidence for gastric cancer according to age, sex, income, region, and CCI.

		N of Event/ N of Total (%)	Follow-Up Duration (PY)	IR per 1000 (PY)	IRD (95%CI)	Hazard Ratios for Osteoporosis			
						Crude †	*p* Value	Adjusted ‡	*p* Value
Total participants (n = 45,390)									
	Gastric cancer	596/9078 (6.57)	45,188	13.20	1.80 (0.73 to 2.91)	1.16 (1.06–1.27)	0.001 *	1.13 (1.03–1.24)	0.01 *
	Control	2602/36,312 (7.17)	228,816	11.40		1		1	
Age < 65 years old (n = 24,765)									
	Gastric cancer	258/4953 (5.21)	29,362	8.79	2.40 (1.36 to 3.43)	1.35 (1.18–1.55)	<0.001 *	1.28 (1.11–1.48)	<0.001 *
	Control	916/19,812 (4.62)	143,267	6.39		1		1	
Age ≥ 65 years old (n = 20,625)									
	Gastric cancer	338/4125 (8.19)	15,826	21.40	1.70 (−0.75 to 4.05)	1.04 (0.93–1.17)	0.47	1.03 (0.91–1.16)	0.643
	Control	1686/16,500 (10.22)	85,549	19.70		1		1	
Male (n = 33,080)									
	Gastric cancer	222/6616 (3.36)	33,453	6.64	1.49 (0.63 to 2.35)	1.31 (1.13–1.52)	<0.001 *	1.24 (1.07–1.45)	0.005 *
	Control	868/26,464 (3.28)	168,563	5.15		1		1	
Female (n = 12,310)									
	Gastric cancer	374/2462 (15.19)	11,735	31.90	3.10 (−0.29 to 6.48)	1.08 (0.97–1.21)	0.173	1.08 (0.96–1.21)	0.196
	Control	1734/9848 (17.61)	60,253	28.80		1		1	
Low-income group (n = 21,720)									
	Gastric cancer	263/4344 (6.05)	20,713	12.70	1.50 (−0.04 to 3.13)	1.13 (0.99–1.29)	0.071	1.11 (0.96–1.27)	0.147
	Control	1207/17,376 (6.95)	108,226	11.20		1		1	
High-income group (n = 23,670)									
	Gastric cancer	333/4734 (7.03)	24,475	13.60	2.00 (0.54 to 3.54)	1.18 (1.05–1.33)	0.007 *	1.14 (1.01–1.30)	0.031 *
	Control	1395/18,936 (7.37)	120,590	11.60		1		1	
Urban resident (n = 19,460)									
	Gastric cancer	225/3892 (5.78)	20,013	11.20	1.00 (−0.49 to 2.60)	1.08 (0.94–1.25)	0.271	1.07 (0.92–1.24)	0.362
	Control	1030/15,568 (6.62)	101,128	10.20		1		1	
Rural resident (n = 25,930)									
	Gastric cancer	371/5186 (7.15)	25,175	14.70	2.40 (0.90 to 3.95)	1.21 (1.08–1.35)	0.001 *	1.17 (1.04–1.31)	0.01 *
	Control	1572/20,744 (7.58)	127,688	12.30		1		1	
CCI scores = 0 (n = 25,004)									
	Gastric cancer	217/3128 (6.94)	17,753	12.20	2.82 (1.31 to 4.38)	1.29 (1.11–1.49)	<0.001 *	1.34 (1.16–1.55)	<0.001 *
	Control	1331/21,876 (6.08)	141,902	9.38					
CCI scores = 1 (n = 7719)									
	Gastric cancer	110/1758 (6.26)	10,064	10.90	−3.30 (−5.82 to −0.70)	0.76 (0.62–0.93)	0.009 *	0.94 (0.76–1.16)	0.553
	Control	530/5961 (8.89)	37,353	14.20					
CCI scores ≥ 2 (n = 12,667)									
	Gastric cancer	269/4192 (6.42)	17,371	15.50	0.50 (−1.59 to 2.66)	0.99 (0.86–1.14)	0.914	1.08 (0.94–1.25)	0.283
	Control	741/8475 (8.74)	49,561	15.00					

Abbreviations: CCI, Charlson Comorbidity Index; IR, incidence rate; IRD, incidence rate difference; PY, person-year; 95%CI, 95% confidence interval. * Stratified and unstratified Cox proportional hazard regression model, significance at *p* < 0.05. † Models were stratified by age, sex, income, and region of residence. ‡ Adjusted for CCI scores.

## Data Availability

Restrictions apply to the availability of these data. The data were obtained from the Korean National Health Insurance Sharing Service (NHISS) and are available at https://nhiss.nhis.or.kr (accessed on 25 January 2022) with the permission of the NHIS.
